# Making Metadata Machine-Readable as the First Step to Providing Findable, Accessible, Interoperable, and Reusable Population Health Data: Framework Development and Implementation Study

**DOI:** 10.2196/56237

**Published:** 2024-08-01

**Authors:** David Amadi, Sylvia Kiwuwa-Muyingo, Tathagata Bhattacharjee, Amelia Taylor, Agnes Kiragga, Michael Ochola, Chifundo Kanjala, Arofan Gregory, Keith Tomlin, Jim Todd, Jay Greenfield

**Affiliations:** 1 Department of Population Health Faculty of Epidemiology and Population Health London School of Hygiene and Tropical Medicine London United Kingdom; 2 African Population and Health Research Center Nairobi Kenya; 3 Malawi University of Business and Applied Science Blantyre Malawi; 4 Independent researcher Lilongwe Malawi; 5 Committee on Data (CODATA) Paris France

**Keywords:** FAIR data principles, metadata, machine-readable metadata, DDI, Data Documentation Initiative, standardization, JSON-LD, JavaScript Object Notation for Linked Data, OMOP CDM, Observational Medical Outcomes Partnership Common Data Model, data science, data models

## Abstract

**Background:**

Metadata describe and provide context for other data, playing a pivotal role in enabling findability, accessibility, interoperability, and reusability (FAIR) data principles. By providing comprehensive and machine-readable descriptions of digital resources, metadata empower both machines and human users to seamlessly discover, access, integrate, and reuse data or content across diverse platforms and applications. However, the limited accessibility and machine-interpretability of existing metadata for population health data hinder effective data discovery and reuse.

**Objective:**

To address these challenges, we propose a comprehensive framework using standardized formats, vocabularies, and protocols to render population health data machine-readable, significantly enhancing their FAIRness and enabling seamless discovery, access, and integration across diverse platforms and research applications.

**Methods:**

The framework implements a 3-stage approach. The first stage is Data Documentation Initiative (DDI) integration, which involves leveraging the DDI Codebook metadata and documentation of detailed information for data and associated assets, while ensuring transparency and comprehensiveness. The second stage is Observational Medical Outcomes Partnership (OMOP) Common Data Model (CDM) standardization. In this stage, the data are harmonized and standardized into the OMOP CDM, facilitating unified analysis across heterogeneous data sets. The third stage involves the integration of Schema.org and JavaScript Object Notation for Linked Data (JSON-LD), in which machine-readable metadata are generated using Schema.org entities and embedded within the data using JSON-LD, boosting discoverability and comprehension for both machines and human users. We demonstrated the implementation of these 3 stages using the Integrated Disease Surveillance and Response (IDSR) data from Malawi and Kenya.

**Results:**

The implementation of our framework significantly enhanced the FAIRness of population health data, resulting in improved discoverability through seamless integration with platforms such as Google Dataset Search. The adoption of standardized formats and protocols streamlined data accessibility and integration across various research environments, fostering collaboration and knowledge sharing. Additionally, the use of machine-interpretable metadata empowered researchers to efficiently reuse data for targeted analyses and insights, thereby maximizing the overall value of population health resources. The JSON-LD codes are accessible via a GitHub repository and the HTML code integrated with JSON-LD is available on the Implementation Network for Sharing Population Information from Research Entities website.

**Conclusions:**

The adoption of machine-readable metadata standards is essential for ensuring the FAIRness of population health data. By embracing these standards, organizations can enhance diverse resource visibility, accessibility, and utility, leading to a broader impact, particularly in low- and middle-income countries. Machine-readable metadata can accelerate research, improve health care decision-making, and ultimately promote better health outcomes for populations worldwide.

## Introduction

Population health data play a crucial role in understanding the dynamics of public health and informing evidence-based policies and interventions [1]. In low- and middle-income countries (LMICs), two common approaches for systematically and continuously monitoring health indicators in the population over time are disease surveillance systems and health and demographic surveillance systems (HDSSs). Surveillance systems are designed to detect and respond to outbreaks of infectious diseases, whereas HDSSs are longitudinal systems that collect health and demographic data for a defined population [2,3].

Despite the availability of valuable population health data from these systems, there remains a significant challenge in effectively sharing this information across research entities. Finding data and knowledge or information about population health requires accurate and comprehensive documentation of metadata, often referred to as “data about data”; however, the best way to achieve this remains unclear [4]. Disease surveillance metadata encompass crucial details, including the diseases under surveillance, geographical areas covered, and variables measured, each with defined formats and explanations. For example, variables may include disease incidence rates, demographic characteristics, and health care utilization. Additionally, a data dictionary provides comprehensive definitions and formats for each variable, aiding in the interpretation and use of surveillance data [5].

Metadata are a critical component of achieving the findable, accessible, interoperable, and reusable (FAIR) data principles [6]. The integration of these principles into data science aligns with the original vision of Wilkinson et al [6], which goes beyond the traditional reuse of data but also other digital research components such as inputs, outputs, algorithms, tools, and workflows that generate data [7]. Realizing this vision requires the use of not only one but several standards, depending on the use case [6]. The FAIRness of a data collection process can be ensured by using metadata standards such as the Data Documentation Initiative (DDI), Dublin Core, and Common Data Model (CDM) for representing exposures and outcomes, along with a domain-specific vocabulary for annotating these standards [8,9].

The DDI Codebook and DDI Lifecycle serve as international standards for systematically detailing data generated through surveys and observational methods, ensuring consistent documentation of content, structure, and provenance. This enhances the accessibility, discovery, and preservation of data and consistency in representation of the data by users [10]. Notable portals such as INDEPTH Data Repository (iSHARE), SAPRIN Data, and the African Population Health Research Center’s microdata portal employ the DDI for public data dissemination [11-13].

The Observational Medical Outcomes Partnership (OMOP) CDM addresses the challenge of integrating and analyzing diverse health care data by providing a standardized information model. This model acts as a universal language, enabling seamless integration and consistent analysis of data from various home and clinic encounters [14,15].

The core of the OMOP CDM lies in its well-defined structure comprising 39 tables categorized into relevant health care domains [16]. These domains include standardized vocabularies, person-centric data (eg, demographics and diagnoses), and standardized health system data (eg, procedures and medications). This organization ensures consistency throughout the data and facilitates downstream analyses [15].

Furthermore, the standardized structure allows efficient data preparation through extraction, transformation, and loading (ETL) processes for analysis with various tools. This facilitates uniform analysis techniques across studies. Additionally, a standardized vocabulary enables domain-specific labeling of interventions and outcomes, which is crucial for machine learning and metadata documentation within the CDM framework [17].

The OMOP CDM prioritizes ethical considerations by sharing deidentified and aggregated data, enabling network-wide analysis without sharing patient-level information. This promotes transparency, reduces bias, and aligns with data protection regulations while enabling autonomous data sharing and safeguarding individual privacy with data remaining secure at the source [18].

While data standardization and harmonization are essential for FAIR compliance, they are not sufficient on their own [6]. Another step involves rendering metadata machine-readable and more findable online, aligning with the objectives of Schema.org, a collaborative project among major search engines such as Google. Schema.org was established to create a schema or shared vocabulary dedicated to developing and establishing metadata standards that enhance the discoverability and indexing of online content, including assets in population health and clinical data [19]. Although Schema.org has applicability across a vast range of domains, its use in population health data remains underexplored. Nevertheless, harnessing Schema.org offers the potential to significantly streamline data discovery efforts, as evidenced by its adoption by over 10 million websites [19].

For this reason, we are considering using JavaScript Object Notation for Linked Data (JSON-LD), a lightweight linked data format capable of marking up internet content with metadata. JSON-LD initially used Schema.org entities such as Action, BioChemEntity, CreativeWork, Event, MedicalEntity, Organization, Person, and Place; however, its capabilities extend beyond these entities. JSON-LD can create complex machine-readable documents that can seamlessly transition between different sets of metadata objects. In our use case and others, JSON-LD demonstrated the potential to FAIRify the arc of data science by enabling seamless interoperability and data sharing across diverse systems and platforms [20].

However, the current practice of FAIR implementation in population health faces substantial barriers. Obstacles include the limited availability of mature FAIR technology, the proliferation of diverse digital tools, and data often being locked within local formats or proprietary standards imposed by electronic health record vendors. Moreover, these challenges are compounded by a prevailing siloed data mindset among the key stakeholders collecting population health data [21].

We here propose an approach to bridge the gap between FAIR principles and practice in population health data by incorporating machine-readable metadata for data and nondata digital assets, including platforms, into the population health data set. We use the Integrated Disease Surveillance and Response (IDSR) data as a case study to demonstrate the proposed processes. Despite its critical role in public health, the current structure of IDSR data presents a challenge in adhering to FAIR principles. Key information regarding data collection methodologies, access restrictions, and updates often lacks proper documentation or resides scattered across diverse formats. This fragmentation severely hinders the findability, accessibility, and interoperability of the data, posing substantial obstacles to its effective use and compliance with FAIR standards. To address this, we leverage established standards such as the DDI and OMOP CDM and explore the potential of Schema.org entities alongside other standards using JSON-LD to extend and adapt these principles. This strategic combination of machine-readable metadata and standards such as Schema.org and others coupled with JSON-LD is a crucial step toward achieving *comprehensive* FAIR compliance in population health data.

The proposed approach integrates the collaborative frameworks of the GO-FAIR and WorldFAIR initiatives to promote interdisciplinary collaboration, culture change, and technology integration for the effective implementation of FAIR principles [22,23]. This effort addresses the long-standing challenge of restricted data discoverability, which hinders the effective use, reuse, integration, and knowledge integration of data [24]. Data stewardship plans mandating the data, along with all assets generated from public funds, should be publicly accessible [25]. Good data stewardship practices, particularly those adhering to FAIR principles, significantly enhance data discoverability and facilitate their improved reuse. Universal access to health research data, regardless of location or resource limitations, is crucial for advancing research and supports the broader goal of promoting healthy lives and well-being for all at all ages, as outlined in Sustainable Development Goal (SDG) 3, for improving global health and achieving SDGs [26,27].

## Methods

### Framework

We propose a step-by-step guide to making metadata FAIR using the IDSR database, including data collected in population settings and HDSS sites in Africa, as a demonstration of our use case. Our methodology is built upon a flexible, multilevel, domain-agnostic FAIRification framework, providing practical guidance to improve the FAIRness for both existing and future data sets. This framework encompasses 3 stages: (1) integration of DDI metadata, (2) implementation of the OMOP CDM, and (3) leveraging of Schema.org to refine the metadata structure and accessibility [28]. We use the IDSR data as a case study, which is recommended by the World Health Organization (WHO) but may be implemented differently from one country to another.

To effectively implement the DDI framework for population health data, we first selected the National Data Archive (NADA) online catalog. This metadata repository adheres to the DDI 2 Codebook and Dublin Core XML metadata standards [29]. NADA serves as a comprehensive platform for searching, comparing, applying for access to, and downloading metadata, data sets, questionnaires, and reports. NADA plays a pivotal role in ensuring the accessibility, discoverability, reusability, and collaboration of population health data. This is achieved by configuring the open-source web-based data cataloging application according to the guidelines provided in the NADA documentation [30].

Nesstar Publisher is used to create the DDI Codebook, a structured and descriptive document that captures essential metadata elements, which integrates the DDI framework into the IDSR data set [31]. With the help of the International Household Survey Network metadata template and the step-by-step guide, we describe the attributes of the IDSR data in the codebook, providing a rich and informative metadata record, including document description, study description, data file description, variable description, and additional materials [32,33]. More advanced versions of the DDI, such as the DDI Lifecycle, have the potential to extend this integration to longitudinal studies such as the HDSS and other cohort studies, accommodating distinct waves or rounds of data collection [34,35].

The Implementation Network for Sharing Population Information from Research Entities (INSPIRE) developed ETL programs to upload the source data and metadata into the OMOP CDM, harmonizing and standardizing the data for unified data analysis in research using open-source Observational Health Data Sciences and Informatics (OHDSI) tools, which extend beyond their conventional use in clinical data [36,37].

This framework builds on the methods introduced by the European Health Data & Evidence Network [38] to grow the description of the IDSR data and metadata as a use case within the OMOP CDM. The process of FAIRification begins by identifying various digital resources present within the OHDSI artifacts, including *protocols*, *databases*, *study results*, *controlled vocabularies*, *software libraries*, and any other relevant digital assets. The metadata are described using Schema.org, expressed in JSON-LD format.

**Figure 1:**
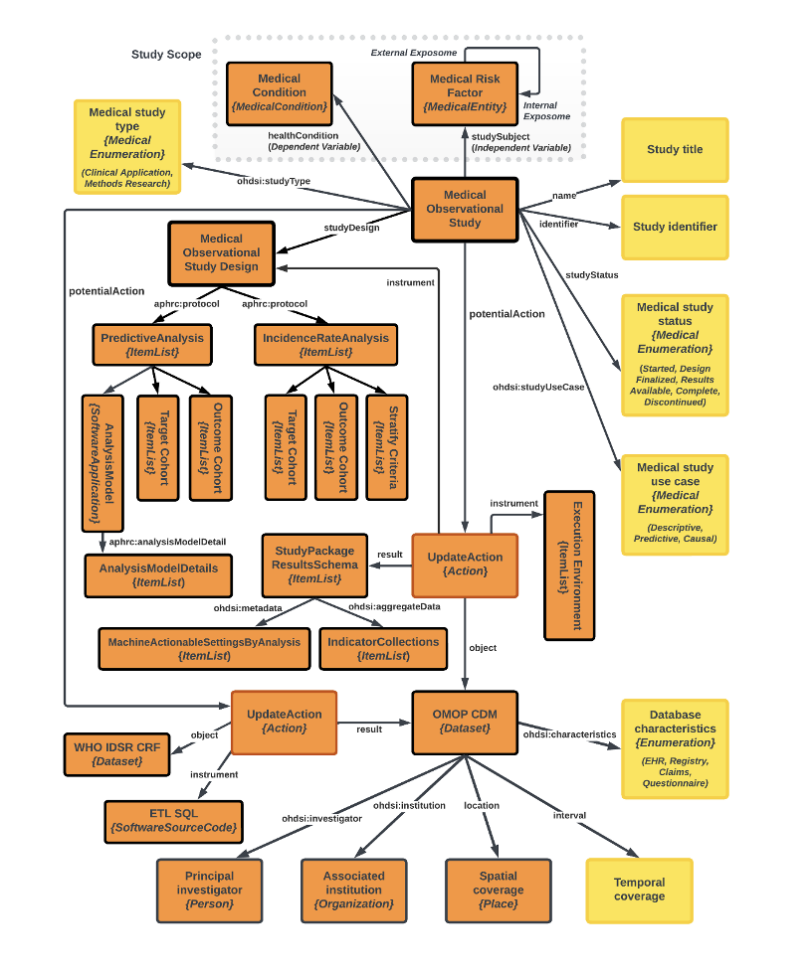
Structure of the Implementation Network for Sharing Population Information from Research Entities (INSPIRE) model. aphrc: African Population Health Research Center; CRF: cloud raster format; EHR electronic health record; ETL: extraction, transform, load; IDSR: Integrated Disease Surveillance and Response; ohdsi: Observational Health Data Sciences and Informatics; OMOP CDM: Observational Medical Outcomes Partnership Common Data Model; WHO: World Health Organization.

The standard CDM has the structure shown in Figure 1. The yellow boxes are literals that describe elements of the Schema.org MedicalObservationalStudy category, including the title, identifier, database, study status, and type. The orange boxes describe a set of classes or concepts that are contained in the data set. These classes use standard vocabulary concepts that describe variables in the study, including the risk factors and exposures (schema: MedicalEntity) and the conditions reported (schema: MedicalCondition) in connection with them. The left of the figure shows the analyses carried out on the data. The lower part of the model outlines the initial step of generating the OMOP CDM instance through an action that uses the ETL process leveraging the Pentaho platform and SQL to integrate and process data from the IDSR source data set or synthetic data.

The validity of the generated JSON-LD files can be verified using the Schema.org validator, a user-friendly tool capable of validating JSON-LD code. Validation can be done by either submitting a URL that points to the JSON-LD file or by directly pasting the JSON-LD code into the tool. The JSON-LD is then embedded within the HTML code, enabling search engines such as Google [39] to effectively use these structured data for enhanced data discovery and comprehension purposes.

### Ethical Considerations

This research centers on implementing the FAIR principles. As there was no involvement of human subjects in our research, ethical approval from a local ethics committee is not applicable.

## Results

### DDI Codebook

The DDI codebook presented through the NADA repository provides a comprehensive and structured documentation framework for the IDSR source data. This resource is essential for researchers seeking a comprehensive understanding of the data set. For the complete IDSR codebook, please refer to Multimedia Appendix 1. The codebook is logically organized into four main sections, as outlined in Table 1.

**Table 1:** Catalog sections of the Data Documentation Initiative (DDI) codebook for Integrated Disease Surveillance and Response (IDSR) source data.

Section	Description
Study description	The Study section of the DDI codebook provides a comprehensive overview of the IDSR study, including its title, purpose, methodology, coverage, producers and sponsors, disclaimer and copyright, and contact details.
Documentation	This section of the DDI codebook includes the World Health Organization IDSR questionnaire for Malawi, Kenya, and Uganda, as well as other relevant documentation for the IDSR study, such as the study protocol.
Data description	Provides a detailed description of the IDSR data set, including the data files from Malawi, Kenya, and Uganda. The codebook provides detailed descriptions of all variables in the data set, including their names, labels, definitions, and coding schemes.
Microdata	This section provides information about the IDSR source data, including the number of variables, their corresponding format, and a description of each variable. The raw microdata are not currently shared, but have been ETLed^a^ into the OMOP CDM^b^ and the results are accessible through ATLAS [40].

^a^ETLed: extracted, transformed, and loaded.

^b^OMOP CDM: Observational Medical Outcomes Partnership Common Data Model.

The detailed description of the IDSR study offers a deep understanding of the data set, ensuring clarity for researchers. Accessible questionnaires further enrich the resource, providing invaluable insights into the data collection process.

The user interface is intuitively designed, offering seamless navigation and search capabilities. Researchers can effortlessly locate codebooks through keyword, title, or author searches. The availability of multiple download formats, including XML, PDF, and HTML, enhances accessibility.

A notable feature is that the support for the repository extends its accessibility through multilingual metadata support, accommodating researchers from diverse linguistic backgrounds. This inclusivity further bolsters the codebook’s utility and accessibility.

Additionally, the codebook maintains a detailed history of versions and updates made to the data set. This feature ensures transparency and aids researchers in understanding potential impacts on their analyses.

### JSON-LD Representation

JSON-LD was used to create a structured representation of the IDSR data. This format leverages the Schema.org vocabulary, a common language for describing things on the internet. Specifically, we used properties from the MedicalObservationalStudy schema to capture essential information about the public health study, such as the study design (eg, case series, cohort, observational, cross-sectional, longitudinal, or registry). Additionally, properties such as schema.org/healthCondition, schema.org/studyLocation, schema.org/studySubject, and schema.org/guideline are used to describe patient population characteristics (eg, inclusion criteria, age range), exposure or outcome measures of interest, and relevant health conditions or guidelines. Furthermore, this approach aligns with the efforts of OHDSI, which has adopted Schema.org as a FAIRifying standard and extended its usage through OHDSI extension.

**Figure 2:**
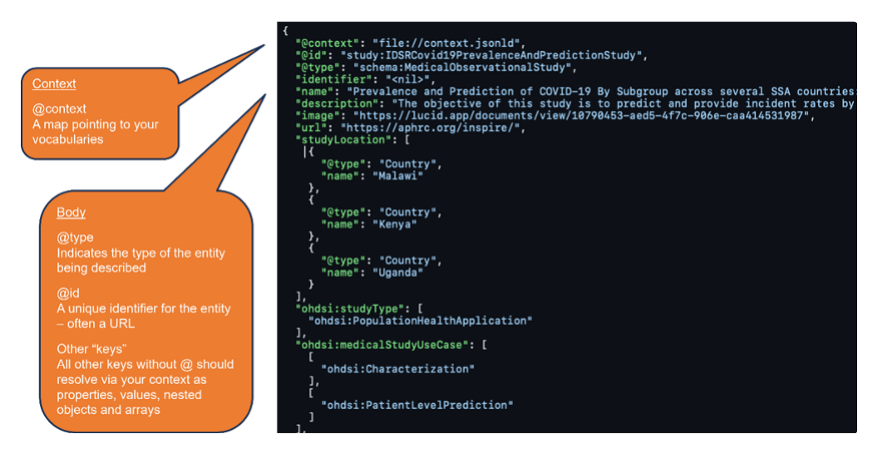
Syntax example: MedicalObservationalStudy.

This structured representation benefits both human users and machine processing. For public health users familiar with Schema.org, the data are easier to understand and interpret. Additionally, the use of a common vocabulary facilitates data exchange and integration with other systems that leverage Schema.org. Figure 2 showcases a snippet of the JSON-LD code, illustrating how specific data elements are mapped to Schema.org properties. Table 2 provides a more detailed tabular view of the IDSR JSON-LD class structure, including corresponding properties and their values.

**Table 2:** Javascript Object Notation–Linked Data (JSON-LD) class description.

Class	Description
MedicalObservationalStudy	This represents the core IDSR^a^ study being described. It includes the Study Title, Identifier, Status, and Use Case.
MedicalRiskFactor	Describes both internal and external exposomes (ie, the exposure of individuals in their environment).
DatabaseCharacteristics	This class describes the IDSR data set, which is implemented using the OMOP CDM^b^ v5.4.4. The IDSR data set is a federated system that includes data from Malawi, Kenya, and Uganda. The data set is stored in a relational database with the following tables: Person, Condition_Occurrence, Observation, Drug_Exposure, Procedure_Occurrence, and Measurement.
MedicalCondition	This class describes the medical conditions in the IDSR data, including their descriptions and OMOP CDM concept IDs.
MedicalObservationalStudyDesign	Provides detailed information on the study’s design, including types of analyses (eg, predictive analysis, incident rate analysis).
UpdateAction	UpdateActions describe the workflow in the MedicalObservationStudy beginning with an action that takes the source data as input (object) and produces an OMOP CDM instance as output (result) using an ETL^c^ SQL as an instrument. A second UpdateAction takes the OMOP CDM as an object and populates the OMOP CDM Results Schema as a result using both the MedicalObservationalStudyDesign and its execution environment as instruments.

^a^ISDR: Integrated Disease Surveillance and Response.

^b^OMOP CM: Observational Medical Outcomes Partnership Common Data Model.

^c^ETL: extraction, transformation, and loading.

Our structured representation methodology makes the IDSR data set easily discoverable through platforms such as Google Dataset Search. The JSON-LD codes are available on the GitHub repository [41]. Additionally, the HTML code, embedded with JSON-LD, can be found on the INSPIRE website [42]. This open-source approach promotes transparency, reproducibility, and further development of our work.

## Discussion

### Prospects and Challenges

The integration of the OMOP CDM, DDI, and Schema.org with JSON-LD provides access to the metadata within the IDSR framework. This led to substantial improvements in the FAIRness, standardization, interoperability, and analytical capabilities of the IDSR data, reinforcing the critical role of machine readability in this domain. Notably, the efficient sharing of vital metadata enables seamless data integration, collaborative research, and advanced analysis across diverse contexts, which is essential for improving public health outcomes. The model may also be used to promote the discoverability, accessibility, and reusability of observational research. One of the main benefits of this integration effort is that IDSR data can become more visible and accessible on the search engine results pages, which can increase the click-through rate [41]. Moreover, sharing nondata research objects such as analytical workflows and code can significantly enrich the research ecosystem by enabling others to replicate, validate, and extend existing findings, fostering transparency and reproducibility.

However, the adoption of Schema.org with JSON-LD in the context of population health data presents certain challenges. Specifically, OHDSI vocabulary predominantly consists of medical terms, lacking comprehensive coverage of population health–related concepts such as HDSSs and IDSRs. Standard vocabularies such as Systematized Nomenclature of Medicine–Clinical Terms (SNOMED-CT) and Logical Observation Identifiers Names and Codes (LOINC) often miss tests and questionnaires common in LMICs, which is particularly evident in capturing stages and public health–clinical interactions for diseases like AIDS. Terminologies such as those of the Columbia International eHealth Laboratory, already integrated into OHDSI, offer significant potential as independent standard vocabularies to address these gaps [43].

To effectively address the specific gaps identified within the MedicalObservationalStudy model for African contexts, it is crucial to focus on enhancing vocabularies tailored to the region. This entails prioritizing risk factors and exposures currently inadequately captured by existing OHDSI standards. Particularly, OHDSI lacks vocabularies encompassing physical/chemical and social determinants of health as well as mental health factors. Our participation in an OHDSI Working Group directly addresses these deficiencies by examining the relationships between diverse exposure histories (eg, climate variations, pharmaceutical accessibility, and health care availability for vulnerable populations such as pregnant women) and the medical conditions presented by study participants.

Importantly, technical expertise is necessary to optimize web pages for search engine optimization, ensuring effective implementation of Schema.org with JSON-LD. This highlights the importance of community support in developing and refining the necessary resources and expertise to overcome these challenges and maximize the benefits of adopting Schema.org with JSON-LD for population health data in Africa.

While the DDI codebook is a valuable tool for metadata documentation, it may not be as effective as the DDI Lifecycle for promoting reuse under FAIR principles, as noted by Kanjala et al [35]. The DDI codebook is a static document that describes the data in a study at a single point in time, whereas the dynamic DDI Lifecycle can be used to describe the data throughout their life cycle, from collection to dissemination. Moreover, the DDI Lifecycle is more machine-actionable, automating tasks such as data validation and interoperability. As a result, adoption of the DDI Lifecycle presents a promising avenue for future research to further enhance the accessibility and reusability of population health data in LMIC contexts.

The study’s findings underscore the substantial benefits of adopting OMOP CDM, DDI, and Schema.org with JSON-LD and suggest that these benefits outweigh the accompanying challenges. However, it is imperative to proactively address these challenges to ensure the successful implementation and adoption of these technologies. By actively tackling the challenges and offering robust support to users, the IDSR or HDSS community can significantly enhance the FAIRness and accessibility of their data and digital assets, enabling a broader spectrum of users to leverage their potential for research, decision-making, and public health interventions.

### Future Directions

In future strategies, the FAIRification process will emphasize the adoption of Schema.org and JSON-LD MedicalObservationStudy as a standard within the Cross-Domain Interoperability Framework. This framework is capable of describing a workflow that includes the WHO IDSR platform, the OMOP CDM, and the OHDSI data analysis workbench. Regardless of whether each locality has its own platform, the data stay at home and only methods and aggregate results are shared or the data are pooled and move through the entire workflow at once. Indeed, because the MedicalObservationalStudy can describe the entire arc of clinical and population health research, it more or less guarantees the reproducibility of studies.

In the future, our aim is to expand the capabilities of MedicalObservationalStudy in several ways. First, we intend for it to describe a workflow that includes climate events and social determinants of health, which is crucial for understanding outcomes [44]. Furthermore, we seek to enhance the MedicalObservationalStudy to describe a workflow that terminates in a data cube in which each cell disaggregates an aggregate result by many dimensions [45] . This would be a workflow that INSPIRE augments with a Statistical Data and Metadata eXchange (SDMX) instance to present the OMOP CDM Results schema as an indicator repository [46]. This addition to the workflow mirrors the United Nations SDG platform and ensures broader utilization.

**Figure 3:**
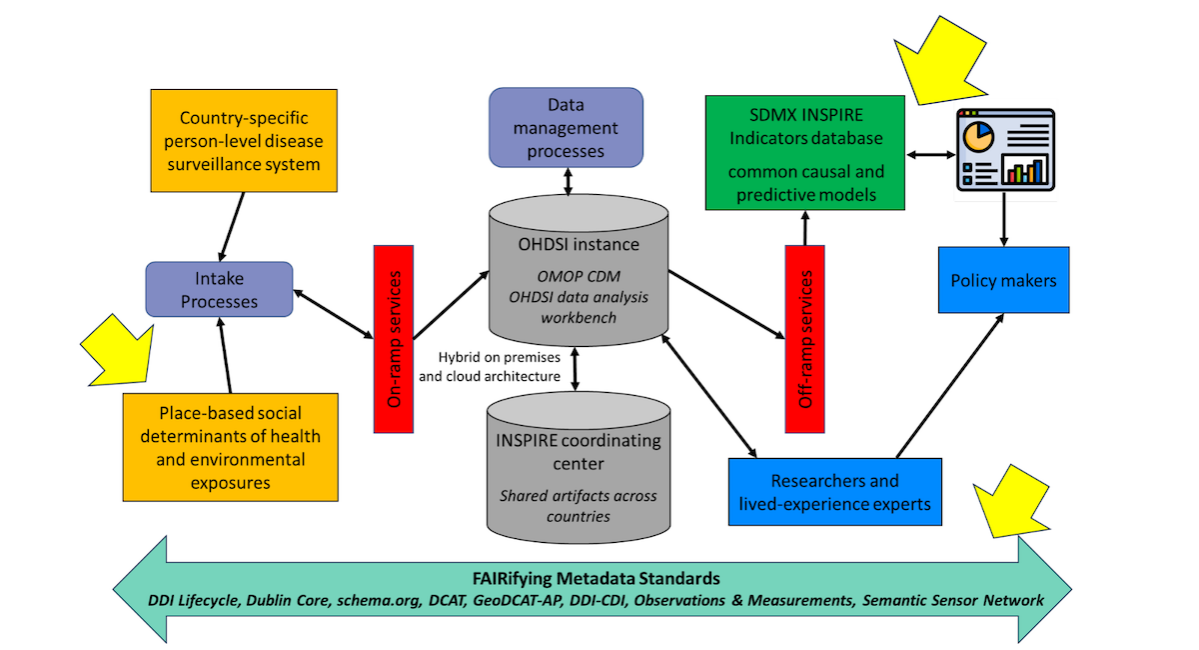
Next steps to achieving machine-readable and machine-actionable metadata for public health. CDI: Comprehensive Data Integration; DCAT: Data Catalog Vocabulary; DDI: Data Documentation Initiative; FAIR: findable, accessible, interoperable, reusable; GeoDCAT-AP: a geospatial extension for the DCAT application profile for data portals in Europe; INSPIRE: Implementation Network for Sharing Population Information with Research Entities; OHDSI: Observational Health Data Sciences and Informatics; OMOP CDM: Observational Medical Outcomes Partnership Common Data Model; SDMX: Statistical Data and Metadata Exchange.

While SDMX is prominent, the DDI–Comprehensive Data Integration offers arguably a more capable format that statistical organizations may adopt in the future [45]. In addition, the ongoing integration of Fast Healthcare Interoperability Resources with OHDSI may provide other paths for a future workflow to follow [47]. Through the navigation of these evolving platforms and their standards, our work with the MedicalObservationalStudy aims to achieve compatibility, relevance, and interoperability within the public health community. The arrows in Figure 3 show where machine-readable and machine-actionable metadata are still needed.

Finally, as new and emerging data formats emerge, Schema.org’s flexibility and extensibility will play a crucial role in accommodating these formats, ensuring continued compatibility and interoperability within the evolving landscape of population health data.

### Conclusions

The use of machine-readable metadata plays a vital role in ensuring the FAIRification of population health data. By embracing universal standards such as those of Schema.org, organizations can not only enhance their search engine optimization but also make their data more discoverable on the internet. This will maximize the impact and utility of population health data, particularly in LMICs. This paper highlights the importance of promoting and adopting machine-readable metadata standards in LMICs to advance the FAIRification and accessibility of population health data. 

## Appendix

Multimedia Appendix 1 Data Documentation Initiative (DDI) documentation.

## Abbreviations

CDM: Common Data Model
DDI: Data Documentation Initiative
ETL: extraction, transform, load
FAIR: findable, accessible, interoperable, reusable
HDSS: health and demographic surveillance system
IDSR: Integrated Disease Surveillance and Response
INSPIRE: Implementation Network for Sharing Population Information with Research Entities
JSON-LD: Javascript Object Notation–Linked Data
LMIC: low- and middle-income country
LOINC: Logical Observation Identifiers Names and Codes
NADA: National Data Archive
OHDSI: Observational Health Data Sciences and Informatics
OMOP: Observational Medical Outcomes Partnership
SDG: Sustainable Development Goal
SDMX: Statistical Data and Metadata Exchange
SNOMED-CT: Systematized Nomenclature of Medicine–Clinical Terms
WHO: World Health Organization
