# Correction: Making Metadata Machine-Readable as the First Step to Providing Findable, Accessible, Interoperable, and Reusable Population Health Data: Framework Development and Implementation Study

**DOI:** 10.2196/65249

**Published:** 2024-08-14

**Authors:** David Amadi, Sylvia Kiwuwa-Muyingo, Tathagata Bhattacharjee, Amelia Taylor, Agnes Kiragga, Michael Ochola, Chifundo Kanjala, Arofan Gregory, Keith Tomlin, Jim Todd, Jay Greenfield

**Affiliations:** 1 Department of Population Health Faculty of Epidemiology and Population Health London School of Hygiene and Tropical Medicine London United Kingdom; 2 African Population and Health Research Center Nairobi Kenya; 3 Malawi University of Business and Applied Science Blantyre Malawi; 4 Independent researcher Lilongwe Malawi; 5 Committee on Data (CODATA) Paris France

In “Making Metadata Machine-Readable as the First Step to Providing Findable, Accessible, Interoperable, and Reusable Population Health Data: Framework Development and Implementation Study” (Online J Public Health Inform 2024;16:e56237) the authors noted one error.

The group author “INSPIRE INSPIRE network” has been removed from the end of the authorship list.

The correction will appear in the online version of the paper on the JMIR Publications website on August 14, 2024, together with the publication of this correction notice. Because this was made after submission to PubMed, PubMed Central, and other full-text repositories, the corrected article has also been resubmitted to those repositories.

