# Supplementary material for: Making Metadata Machine-Readable as the First Step to Providing Findable, Accessible, Interoperable, and Reusable Population Health Data: Framework Development and Implementation Study
Source: Online J Public Health Inform. 2024 Aug 1;16:e56237. doi: 10.2196/56237 (PMC11327634; doi:10.2196/56237)
Supplement: Multimedia Appendix 1 [file ojphi_v16i1e56237_app1.pdf]

Malawi, Uganda, Kenya - A Platform for Evaluation and Analysis of COVID-19 Harmonised data (PEACH): Metadata

Dr Amelia Taylor, Dr Sylvia Muyingo, Prof Jim Todd, Public Health Institute of Malawi

Report generated on: September 30, 2023

Visit our data catalog at: http://51.105.33.160/microdata/index.php

Overview

Identification

ID NUMBER
INSPIRE.PEACH.COVID.2022.V1

Version

VERSION DESCRIPTION
INSPIRE.PEACH.V1.0 Metadata for public distribution.

Overview

ABSTRACT
The emergence of COVID-19 as a global pandemic has posed a critical health threat to numerous low-and-middle-income countries (LMICs) and the well-being of their populations. Timely and precise data are imperative for adapting health policies and strategies to effectively combat this threat. However, obtaining such data is a challenge, particularly under lockdown restrictions, necessitating innovative approaches to data collection and aggregation.

Leveraging Artificial Intelligence (AI) and Data Science (DS) innovations is paramount to acquire accurate, real-time data from diverse sources in LMICs. Additionally, addressing methodological gaps in data integration and enhancing information and research capacity is essential for informed decision-making and effective public health policy formulation.

Integrated Disease Surveillance and Response (IDSR) is a pivotal strategy for the early detection and efficient management of infectious disease outbreaks. It entails the systematic collection, analysis, and dissemination of surveillance data from diverse sources. Endorsed by the World Health Organization (WHO) and the Centers for Disease Control and Prevention (CDC), IDSR is globally utilized to proactively prevent, detect, and address public health threats.

In Malawi, Kenya and Uganda, IDSR program is implemented by the Ministry of Health. The data collected can include information on cases and deaths, risk factors, and transmission patterns, as well as information on public health measures such as testing, contact tracing, and quarantine. The IDSR Case Based Reporting Form may vary depending on the specific needs and resources of each country.

The Implementation Network for Sharing Population Information from Research Entities (INSPIRE) is a global initiative aimed at improving public health by facilitating the sharing of population-level health data among researchers and public health authorities. INSPIRE has build a data hub for standardizing and harmonizing IDSR COVID-19 data and implemented the Observational Medical Outcomes Partnership (OMOP) using a Common Data Model (CDM). OMOP CDM facilitates comprehensive analysis across multiple datasets and support sharing of FAIR (Findable, Accessible, Interoperable, Reusable) data which will be used for evidence-informed policy decision making.

TOPICS

Topic	Vocabulary	URI
COVID-19	MeSH	http://www.ncbi.nlm.nih.gov/mesh
SARS-CoV-2	MeSH	http://www.ncbi.nlm.nih.gov/mesh
COVID-19 Vaccines	MeSH	http://www.ncbi.nlm.nih.gov/mesh
Transmission	MeSH	http://www.ncbi.nlm.nih.gov/mesh
Specimen	MeSH	http://www.ncbi.nlm.nih.gov/mesh
Diagnosis	MeSH	http://www.ncbi.nlm.nih.gov/mesh
pre-existing condition	MeSH	http://www.ncbi.nlm.nih.gov/mesh
Symptom(s)	MeSH	http://www.ncbi.nlm.nih.gov/mesh

Coverage

GEOGRAPHIC COVERAGE

This project will exploit data from three African countries - Kenya, Malawi and Ugandai to develop a scalable, coordinated COVID-19 data ecosystem. The three countries adopted different strategies in combating the pandemic

Producers and Sponsors

PRIMARY INVESTIGATOR(S)

Name	Affiliation
Dr Amelia Taylor	MUBAS
Dr Sylvia Muyingo	APHRC
Prof Jim Todd	LSHTM
Public Health Institute of Malawi	Public Health Institute of Malawi

OTHER PRODUCER(S)

Name	Affiliation	Role
David Amadi	LSHTM	DDI author
Amelia Taylor	MUBAS	Technical Assistance
Tathagata Bhattacharjee	LSHTM	Technical Assistance

FUNDING

Name	Abbreviation	Role
International Development Research Centre	IDRC Global South AI4COVID Program	Current Funder

OTHER ACKNOWLEDGEMENTS

Name	Affiliation	Role
Public Health Institute of Malawi	Malawi MOH	Providing Data
Malawi University of Business and Applied Sciences	MUBAS	Providing IT Infrastucture for Data Processing
African Population and Health Research Center	APHRC	Providing IT Infrastucture for Data Processing

Metadata Production

METADATA PRODUCED BY

Name	Abbreviation	Affiliation	Role
Amelia Taylor	AT	MUBAS	Documentation of Study and Review of the metadata
Jim Todd	JT	LSHTM	Documentation of Study and Review of the metadata
Sylvia Muyingo	JT	APHRC	Documentation of Study and Review of the metadata
David Amadi	DA	LSHTM	Documentation of Study and Review of the metadata

DDI DOCUMENT ID

DDI.INSPIRE.PEACH.2022.V1.0

Sampling

No content available

Questionnaires

No content available

Data Collection

Data Processing

No content available

Data Appraisal

No content available

File Description

Variable List

INSPIRE.PEACH.IDSR.MW.2021.CDM.V1

Content	This dataset is based on the IDSR Case Based Reporting Form used to record COVID-19 cases and tests by the Ministry of Health in Malawi. The form can be found in the archive for the dataset. This form is based on the general IDSR Case Based form in use in Malawi by adding COVID-19 specific fields from the WHO CRF for COVID-19. The development of the dataset was done at the Malawi University of Business and Applied Sciences to be used for the INSPIRE PEACH project. INSPIRE PEACH aims at building a data hub for standardizing and harmonizing Covid-19 data from different sources in Malawi, Uganda and Kenya using the OMOP CDM.
Cases	0
Variable(s)	51
Structure	Type:<br>Keys: ()
Version	
Producer	David Amadi, London School of Hygiene & Tropical Medicine (LSHTM) for INSPIRE Network (https://inspiredata.network/)
Missing Data	

Variables

ID	NAME	LABEL	TYPE	FORMAT	QUESTION
V1	REPR_FACI	Reporting health facility	discrete	numeric	
V2	REPR_DIST	Reporting district	discrete	numeric	
V3	TYP_CASE	Type of hospital visit	discrete	numeric	
V4	REPR_NM	Reporter name	discrete	character	
V5	REPR_PHON_NUM	Reporting phone number	discrete	numeric	
V6	REPR_D	Reporting date	discrete	character	
V7	TYP_REPR_DISE_COND	Type of reporting disease condition	discrete	numeric	
V8	LN_CASE	Last name of case	discrete	character	
V9	FN_CASE	First name of case	discrete	character	
V10	DOB	Date of birth	discrete	character	
V11	NATL	Nationalities	discrete	numeric	
V12	CASE_UID1	Type of identification document	discrete	numeric	
V13	CASE_UID2	Personal identification detail	discrete	character	
V14	DIST_CASE_RESI	District in Malawi where case resides	discrete	numeric	
V15	DIST_CASE_RESI_TYP	Type of residence in the district	discrete	numeric	
V16	SEX	Sex	discrete	numeric	
V17	OCCU	Occupation	discrete	numeric	
V18	PHYS_ADDR	Physical address	discrete	character	
V19	NEAR_LMK	Nearest landmark	discrete	character	
V20	PHON_NUM_CASE	Phone number of case	discrete	numeric	
V21	PARE_CARET_NM	Parent or Caretaker name	discrete	character	
V22	D_SEEN_FACI	Date seen at facility	discrete	character	
V23	VACC	Type of vaccination	discrete	numeric	
V24	VACC_TYP_VAC	Name of covid-19 vaccine	discrete	numeric	

V25	VACC_NUM_DOSE	Number of covid-19 vaccine doses received	discrete	numeric
V26	D_FACI_NOTI_DIST	Date facility notified district	discrete	character
V27	D_L_VACCI	Date of last vaccination	discrete	character
V28	RECE_TRAV_HIST	Recent travel history	discrete	numeric
V29	D_O_RETU	Date case returned home after traveling	discrete	character
V30	ANY_CONT_OT_CASE	Any contact with OT case	discrete	numeric
V31	ANY_CLUSTER	Type of clustering	discrete	numeric
V32	D_ONSE	Date of onset ofthe symptoms	discrete	character
V33	PREG_CASE_FEM	Pregnancy (If case is female)	discrete	numeric
V34	TRIM	The trimester of the pregnancy	discrete	numeric
V35	PRES_SYMP	Presenting symptoms	discrete	numeric
V36	U_CONDS	Underlying conditions	discrete	numeric
V37	COVI_HIST	Covid(+)Hist	discrete	numeric
V38	P_COMPL_FRM_NM	Personal completer Form(Name)	discrete	character
V39	D_S_COLL	Date specimen collected	discrete	character
V40	D_S_SENT_LAB	Date specimen sent to lab	discrete	character
V41	S_TYP	Type of specimen	discrete	numeric
V42	S_COND	Specimen condition	discrete	numeric
V43	D_LAB_RECE_S	Date lab received specimen	discrete	character
V44	TYP_O_TESTS_PERF	Type of covid test	discrete	numeric
V45	TEST_PLAT	Type of covid testing platform	discrete	numeric
V46	FIN_LAB_RESU	Final laboratory result	discrete	numeric
V47	D_LAB_SENT_RESU_DIST	Date lab sent result to District	discrete	character
V48	D_RESU_SENT_HCW	Date result sent to HCW	discrete	character
V49	D_DIST_RECE_RESU	Date district received result	discrete	character
V50	CASE_F_O	Case final outcome	discrete	numeric
V51	CASE_F_CLASS	Case final classification	discrete	numeric

INSPIRE.PEACH.IDSR.UG.2021.CDM.V1

Content	This dataset is based on the IDSR Case Based Reporting Form used to record COVID-19 cases and tests by the Ministry of Health in Uganda. The form can be found in the archive for the dataset. This form is based on the general IDSR Case Based form in use in Uganda by adding COVID-19 specific fields from the WHO CRF for COVID-19.
Cases	0
Variable(s)	45
Structure	Type:<br>Keys: ()
Version	
Producer	David Amadi, London School of Hygiene & Tropical Medicine (LSHTM) for INSPIRE Network (https://inspiredata.network/)
Missing Data	

Variables

ID	NAME	LABEL	TYPE	FORMAT	QUESTION
V52	D_S_COLL	Date specimen collected	discrete	character	
V53	LAB_ID	Unique laboratory identifier	discrete	numeric	
V54	P_PRI_LEV	Patient prioritization level	discrete	numeric	
V55	B_CORD	Bar Code	discrete	numeric	
V56	Serial	Serial number	discrete	numeric	
V57	S_COLL_D	Sample swabbing district	discrete	character	
V58	S_COLL	Sample collection place	discrete	numeric	
V59	H_FACI	Reporting facility	discrete	character	
V60	P_ENT	Point of entry	discrete	character	
V61	W_TST	Who is being tested	discrete	numeric	
V62	TRAV	Traveler going in or out of uganda	discrete	numeric	
V63	R_HW_TST	Reason for health worker test	discrete	numeric	
V64	HW_FAC	Health worker facility	discrete	character	
V65	IQ_D_TST	Isolated/Quarantined testing date	discrete	numeric	
V66	DOB	Date of birth	discrete	character	
V67	AGE_Y	Estimated age in years	discrete	numeric	
V68	AGE_M	Estimated age in months	discrete	numeric	
V69	SEX	sex	discrete	numeric	
V70	NATL	Nationality	discrete	numeric	
V71	SUB_COUNT	Reporting sub county	discrete	numeric	
V72	REPR_DIST	Reporting district	discrete	numeric	
V73	TRK_DEST	Truck destination	discrete	character	
V74	VAC	Vaccinated vaccinated against covid-19	discrete	numeric	
V75	VACC_TYP_VAC	Name of covid-19 vaccine	discrete	numeric	
V76	N_DOSE	Number of doses	discrete	numeric	
V77	D_LST_DOSE	Date of last dose	discrete	character	

V78	P_SYMP	Was patient symptomatic	discrete	numeric
V79	D_ONSE	Date of first symptoms	discrete	character
V80	PRES_SYMP	Presenting symptoms	discrete	numeric
V81	U_CONDS	Underlying conditions	discrete	numeric
V82	U_CONDS_SPEC	Specific underlying conditions	discrete	numeric
V83	S_TYP	Specimen type	discrete	numeric
V84	AG	Date specimen collected	discrete	numeric
V85	T_S_COLL	Time Specimen collected	discrete	numeric
V86	T_RQST	Test requested	discrete	character
V87	S_REFER	Was specimen referred?	discrete	numeric
V88	TST_NM	Tester's name	discrete	character
V89	T_TYP	Type of test	discrete	numeric
V90	FIN_LAB_RES	Final laboratory result	discrete	numeric
V91	D_RES	Date result released	discrete	character
V92	T_RES	Time result released	discrete	numeric
V93	RMK	Remarks	discrete	character
V94	A_TST_R	Additional test required	discrete	numeric
V95	A_TST_T	Additional test type	discrete	numeric
V96	A_TST_D	Additional test date	discrete	character

INSPIRE.PEACH.IDSR.KE.2021.CDM.V1

Content	This dataset is based on the IDSR Case Based Reporting Form used to record COVID-19 cases and tests by the Ministry of Health in Kenya. The form can be found in the archive for the dataset. This form is based on the general IDSR Case Based form in use in Kenya by adding COVID-19 specific fields from the WHO CRF for COVID-19
Cases	0
Variable(s)	56
Structure	Type:<br>Keys: ()
Version	
Producer	David Amadi, London School of Hygiene & Tropical Medicine (LSHTM) for INSPIRE Network (https://inspiredata.network/)
Missing Data	

Variables

ID	NAME	LABEL	TYPE	FORMAT	QUESTION
V97	EPID_NO	Unique identifier	discrete	numeric	
V98	NATL	Nationality	discrete	numeric	
V99	D_F_REC	Date form received at national level	discrete	character	
V100	H_FACI	Reporting health facility	discrete	character	
V101	T_H_FACI	Type of Health facility	discrete	character	
V102	SUB_COUNT	Reporting sub county	discrete	numeric	
V103	COUNT	Reporting county	discrete	numeric	
V104	TYP_REPR_DISE_COND	Type of reporting disease condition	discrete	numeric	
V105	SEX	sex	discrete	numeric	
V106	DOB	Date of birth	discrete	character	
V107	AGE_Y	Estimated age in years	discrete	numeric	
V108	AGE_M	Estimated age in months	discrete	numeric	
V109	AGE_D	Estimated age in days	discrete	numeric	
V110	RES	residence	discrete	numeric	
V111	D_ONSE	Date of onset of illness	discrete	character	
V112	D_SEEN_FACI	Date seen at facility	discrete	character	
V113	D_NOT_SUBC	Date Health Facility Notified sub county level	discrete	character	
V114	HOSP	Hospitalized	discrete	numeric	
V115	D_ADM	Date of admission	discrete	character	
V116	IP_OP_NO	Inpatient/outpatient number	discrete	numeric	
V117	DIAG	Diagnosis	discrete	character	
V118	M_DIAG	Means of Diagnosis	discrete	numeric	
V119	VACC_HIST	Vaccination History	discrete	numeric	
V120	N_DOSE	Number of doses	discrete	numeric	
V121	VACC_L_T_MON	Vaccination given last two months	discrete	numeric	
V122	D_VACC_L_T_MON	Date vaccination given last two months	discrete	character	

V123	CASE_F_CLASS	Status of the Patient	discrete	numeric
V124	D_ONSE_PARA	Date of onset of paralysis	discrete	character
V125	PARA_SYMP	Paralysis signs and symptoms	discrete	numeric
V126	PARA_SYMP_PRES	Paralysis resenting symptoms	discrete	numeric
V127	SITE_PARA	Site of paralysis	discrete	numeric
V128	DELV	Where was the baby delivered	discrete	numeric
V129	H_FACI_DELV	Health facility baby was delivered	discrete	character
V130	CORD_ST	Was cord cut with sterile blade	discrete	numeric
V131	CORD_TREAT	How was cord stump treated	discrete	character
V132	AGE_D_SYMP	how old in days symptoms began	discrete	numeric
V133	SUCK_N	Did baby suck normally at birth	discrete	numeric
V134	N_TT	Case confirmed neonatal tetanus	discrete	numeric
V135	TX_HC	Treated at health facility	discrete	numeric
V136	MOM_ALIVE	Is the mother alive	discrete	numeric
V137	CR_MOM	Did case response for the mother take place	discrete	numeric
V138	CR_COMM	Case response in community	discrete	numeric
V139	FEVER	Presence of fever	discrete	numeric
V140	D_ONSE_RASH	Date of onset of rash	discrete	character
V141	TYP_RASH	Type of rash	discrete	numeric
V142	CONT_INVE	Patient visited for contact investigation	discrete	numeric
V143	D_CONT_INVE	Date of contact investigation	discrete	character
V144	LAB_CON_CASE	Case epidemiologically linked to lab confirmed case	discrete	numeric
V145	S_COLL	Was specimen collected	discrete	numeric
V146	D_S_COLL	Date Specimen collected	discrete	character
V147	S_TYP	Specimen Type	discrete	numeric
V148	D_S_SENT_LAB	Date Specimen sent to lab	discrete	character
V149	NAME_LAB	Name of lab	discrete	character
V150	LAB_RESU_R	Received lab results	discrete	numeric
V151	LAB_RESU_NR	Received lab results not received	discrete	numeric
V152	FIN_LAB_RES	Final laboratory result	discrete	numeric

Reporting health facility (REPR_FACI)
File: INSPIRE.PEACH.IDSR.MW.2021.CDM.V1

Overview

Type: Discrete
Format: numeric
Width: 10
Decimals: 0
Range: 123-123

Valid cases: 0
Invalid: 0

Reporting district (REPR_DIST)
File: INSPIRE.PEACH.IDSR.MW.2021.CDM.V1

Overview

Type: Discrete
Format: numeric
Width: 10
Decimals: 0
Range: 123-123

Valid cases: 0
Invalid: 0

Type of hospital visit (TYP_CASE)
File: INSPIRE.PEACH.IDSR.MW.2021.CDM.V1

Overview

Type: Discrete
Format: numeric
Width: 10
Decimals: 0
Range: 123-123

Valid cases: 0
Invalid: 0

Reporter name (REPR_NM)
File: INSPIRE.PEACH.IDSR.MW.2021.CDM.V1

Overview

Type: Discrete
Format: character
Width: 3

Valid cases: 0
Invalid: 0

Reporting phone number (REPR_PHON_NUM)
File: INSPIRE.PEACH.IDSR.MW.2021.CDM.V1

Overview

Type: Discrete
Format: numeric
Width: 10
Decimals: 0
Range: 123-123

Valid cases: 0
Invalid: 0

Reporting date (REPR_D)
File: INSPIRE.PEACH.IDSR.MW.2021.CDM.V1

Overview

Type: Discrete
Format: character
Width: 11

Valid cases: 0

Type of reporting disease condition (TYP_REPR_DISE_COND)
File: INSPIRE.PEACH.IDSR.MW.2021.CDM.V1

Overview

Type: Discrete
Format: numeric
Width: 10
Decimals: 0
Range: 123-123

Valid cases: 0
Invalid: 0

Last name of case (LN_CASE)
File: INSPIRE.PEACH.IDSR.MW.2021.CDM.V1

Overview

Type: Discrete
Format: character
Width: 3

Valid cases: 0
Invalid: 0

First name of case (FN_CASE)
File: INSPIRE.PEACH.IDSR.MW.2021.CDM.V1

Overview

Type: Discrete
Format: character
Width: 3

Valid cases: 0
Invalid: 0

Date of birth (DOB)
File: INSPIRE.PEACH.IDSR.MW.2021.CDM.V1

Overview

Type: Discrete
Format: character
Width: 11

Valid cases: 0

Nationalities (NATL)
File: INSPIRE.PEACH.IDSR.MW.2021.CDM.V1

Overview

Type: Discrete
Format: numeric
Width: 10
Decimals: 0
Range: 123-123

Valid cases: 0
Invalid: 0

Type of identification document (CASE_UID1)
File: INSPIRE.PEACH.IDSR.MW.2021.CDM.V1

Overview

Type: Discrete
Format: numeric
Width: 10
Decimals: 0
Range: 123-123

Valid cases: 0
Invalid: 0

Personal identification detail (CASE_UID2)
File: INSPIRE.PEACH.IDSR.MW.2021.CDM.V1

Overview

Type: Discrete
Format: character
Width: 3

Valid cases: 0
Invalid: 0

District in Malawi where case resides (DIST_CASE_RESI)
File: INSPIRE.PEACH.IDSR.MW.2021.CDM.V1

Overview

Type: Discrete
Format: numeric
Width: 10
Decimals: 0
Range: 123-123

Valid cases: 0
Invalid: 0

Type of residence in the district (DIST_CASE_RESI_TYP)
File: INSPIRE.PEACH.IDSR.MW.2021.CDM.V1

Overview

Type: Discrete
Format: numeric
Width: 10
Decimals: 0
Range: 123-123

Valid cases: 0
Invalid: 0

Sex (SEX)
File: INSPIRE.PEACH.IDSR.MW.2021.CDM.V1

Overview

Type: Discrete
Format: numeric
Width: 10
Decimals: 0
Range: 1-123

Valid cases: 0
Invalid: 0

Occupation (OCCU)
File: INSPIRE.PEACH.IDSR.MW.2021.CDM.V1

Overview

Type: Discrete
Format: numeric
Width: 10
Decimals: 0
Range: 123-123

Valid cases: 0
Invalid: 0

Physical address (PHYS_ADDR)
File: INSPIRE.PEACH.IDSR.MW.2021.CDM.V1

Overview

Type: Discrete
Format: character
Width: 3

Valid cases: 0
Invalid: 0

Nearest landmark (NEAR_LMK)
File: INSPIRE.PEACH.IDSR.MW.2021.CDM.V1

Overview

Type: Discrete
Format: character
Width: 3

Valid cases: 0
Invalid: 0

Phone number of case (PHON_NUM_CASE)
File: INSPIRE.PEACH.IDSR.MW.2021.CDM.V1

Overview

Type: Discrete
Format: numeric
Width: 10
Decimals: 0
Range: 123-123

Valid cases: 0
Invalid: 0

Parent or Caretaker name (PARE_CARET_NM)
File: INSPIRE.PEACH.IDSR.MW.2021.CDM.V1

Overview

Type: Discrete
Format: character
Width: 3

Valid cases: 0
Invalid: 0

Date seen at facility (D_SEEN_FACI)
File: INSPIRE.PEACH.IDSR.MW.2021.CDM.V1

Overview

Type: Discrete
Format: character
Width: 11

Valid cases: 0

Type of vaccination (VACC)
File: INSPIRE.PEACH.IDSR.MW.2021.CDM.V1

Overview

Type: Discrete
Format: numeric
Width: 10
Decimals: 0
Range: 123-123

Valid cases: 0
Invalid: 0

Name of covid-19 vaccine (VACC_TYP_VAC)
File: INSPIRE.PEACH.IDSR.MW.2021.CDM.V1

Overview

Type: Discrete
Format: numeric
Width: 10
Decimals: 0
Range: 123-123

Valid cases: 0
Invalid: 0

Number of covid-19 vaccine doses received (VACC_NUM_DOSE)
File: INSPIRE.PEACH.IDSR.MW.2021.CDM.V1

Overview

Type: Discrete
Format: numeric
Width: 10
Decimals: 0
Range: 123-123

Valid cases: 0
Invalid: 0

Date facility notified district (D_FACI_NOTI_DIST)
File: INSPIRE.PEACH.IDSR.MW.2021.CDM.V1

Overview

Type: Discrete
Format: character
Width: 11

Valid cases: 0

Date of last vaccination (D_L_VACCI)
File: INSPIRE.PEACH.IDSR.MW.2021.CDM.V1

Overview

Type: Discrete
Format: character
Width: 11

Valid cases: 0

Recent travel history (RECE_TRAV_HIST)
File: INSPIRE.PEACH.IDSR.MW.2021.CDM.V1

Overview

Type: Discrete
Format: numeric
Width: 10
Decimals: 0
Range: 123-123

Valid cases: 0
Invalid: 0

Date case returned home after traveling (D_O_RETU)
File: INSPIRE.PEACH.IDSR.MW.2021.CDM.V1

Overview

Type: Discrete
Format: character
Width: 11

Valid cases: 0

Any contact with OT case (ANY_CONT_OT_CASE)
File: INSPIRE.PEACH.IDSR.MW.2021.CDM.V1

Overview

Type: Discrete
Format: numeric
Width: 10
Decimals: 0
Range: 123-123

Valid cases: 0
Invalid: 0

Type of clustering (ANY_CLUSTER)
File: INSPIRE.PEACH.IDSR.MW.2021.CDM.V1

Overview

Type: Discrete
Format: numeric
Width: 10
Decimals: 0
Range: 123-123

Valid cases: 0
Invalid: 0

Date of onset ofthe symptoms (D_ONSE)
File: INSPIRE.PEACH.IDSR.MW.2021.CDM.V1

Overview

Type: Discrete
Format: character
Width: 11

Valid cases: 0

Pregnancy (If case is female) (PREG_CASE_FEM)
File: INSPIRE.PEACH.IDSR.MW.2021.CDM.V1

Overview

Type: Discrete
Format: numeric
Width: 10
Decimals: 0
Range: 123-123

Valid cases: 0
Invalid: 0

The trimester of the pregnancy (TRIM)
File: INSPIRE.PEACH.IDSR.MW.2021.CDM.V1

Overview

Type: Discrete
Format: numeric
Width: 10
Decimals: 0
Range: 123-123

Valid cases: 0
Invalid: 0

Presenting symptoms (PRES_SYMP)
File: INSPIRE.PEACH.IDSR.MW.2021.CDM.V1

Overview

Type: Discrete
Format: numeric
Width: 10
Decimals: 0
Range: 123-123

Valid cases: 0
Invalid: 0

Underlying conditions (U_CONDS)
File: INSPIRE.PEACH.IDSR.MW.2021.CDM.V1

Overview

Type: Discrete
Format: numeric
Width: 10
Decimals: 0
Range: 123-123

Valid cases: 0
Invalid: 0

Covid(+)Hist (COVI_HIST)
File: INSPIRE.PEACH.IDSR.MW.2021.CDM.V1

Overview

Type: Discrete
Format: numeric
Width: 10
Decimals: 0
Range: 123-123

Valid cases: 0
Invalid: 0

Personal completer Form(Name) (P_COMPL_FRM_NM)
File: INSPIRE.PEACH.IDSR.MW.2021.CDM.V1

Overview

Type: Discrete
Format: character
Width: 3

Valid cases: 0
Invalid: 0

Date specimen collected (D_S_COLL)
File: INSPIRE.PEACH.IDSR.MW.2021.CDM.V1

Overview

Type: Discrete
Format: character
Width: 11

Valid cases: 0

Date specimen sent to lab (D_S_SENT_LAB)
File: INSPIRE.PEACH.IDSR.MW.2021.CDM.V1

Overview

Type: Discrete
Format: character
Width: 11

Valid cases: 0

Type of specimen (S_TYP)
File: INSPIRE.PEACH.IDSR.MW.2021.CDM.V1

Overview

Type: Discrete
Format: numeric
Width: 10
Decimals: 0
Range: 123-123

Valid cases: 0
Invalid: 0

Specimen condition (S_COND)
File: INSPIRE.PEACH.IDSR.MW.2021.CDM.V1

Overview

Type: Discrete
Format: numeric
Width: 10
Decimals: 0
Range: 123-123

Valid cases: 0
Invalid: 0

Date lab received specimen (D_LAB_RECE_S)
File: INSPIRE.PEACH.IDSR.MW.2021.CDM.V1

Overview

Type: Discrete
Format: character
Width: 11

Valid cases: 0

Type of covid test (TYP_O_TESTS_PERF)
File: INSPIRE.PEACH.IDSR.MW.2021.CDM.V1

Overview

Type: Discrete
Format: numeric
Width: 10
Decimals: 0
Range: 123-123

Valid cases: 0
Invalid: 0

Type of covid testing platform (TEST_PLAT)
File: INSPIRE.PEACH.IDSR.MW.2021.CDM.V1

Overview

Type: Discrete
Format: numeric
Width: 10
Decimals: 0

Valid cases: 0
Invalid: 0

Final laboratory result (FIN_LAB_RESU)
File: INSPIRE.PEACH.IDSR.MW.2021.CDM.V1

Overview

Type: Discrete
Format: numeric
Width: 10
Decimals: 0
Range: 123-123

Valid cases: 0
Invalid: 0

Date lab sent result to District (D_LAB_SENT_RESU_DIST)
File: INSPIRE.PEACH.IDSR.MW.2021.CDM.V1

Overview

Type: Discrete
Format: character
Width: 11

Valid cases: 0

Date result sent to HCW (D_RESU_SENT_HCW)
File: INSPIRE.PEACH.IDSR.MW.2021.CDM.V1

Overview

Type: Discrete
Format: character
Width: 11

Valid cases: 0

Date district received result (D_DIST_RECE_RESU)
File: INSPIRE.PEACH.IDSR.MW.2021.CDM.V1

Overview

Type: Discrete
Format: character
Width: 11

Valid cases: 0

Case final outcome (CASE_F_O)
File: INSPIRE.PEACH.IDSR.MW.2021.CDM.V1

Overview

Type: Discrete
Format: numeric
Width: 10
Decimals: 0
Range: 123-123

Valid cases: 0
Invalid: 0

Case final classification (CASE_F_CLASS)
File: INSPIRE.PEACH.IDSR.MW.2021.CDM.V1

Overview

Type: Discrete
Format: numeric
Width: 10
Decimals: 0
Range: 123-123

Valid cases: 0
Invalid: 0

Date specimen collected (D_S_COLL)
File: INSPIRE.PEACH.IDSR.UG.2021.CDM.V1

Overview

Type: Discrete
Format: character
Width: 11

Valid cases: 0

Unique laboratory identifier (LAB_ID)
File: INSPIRE.PEACH.IDSR.UG.2021.CDM.V1

Overview

Type: Discrete
Format: numeric
Width: 10
Decimals: 0
Range: 123-123

Valid cases: 0
Invalid: 0

Patient prioritization level (P_PRI_LEV)
File: INSPIRE.PEACH.IDSR.UG.2021.CDM.V1

Overview

Type: Discrete
Format: numeric
Width: 10
Decimals: 0
Range: 123-123

Valid cases: 0
Invalid: 0

Bar Code (B_CORD)
File: INSPIRE.PEACH.IDSR.UG.2021.CDM.V1

Overview

Type: Discrete
Format: numeric
Width: 10
Decimals: 0

Valid cases: 0
Invalid: 0

Serial number (Serial)
File: INSPIRE.PEACH.IDSR.UG.2021.CDM.V1

Overview

Type: Discrete
Format: numeric
Width: 10
Decimals: 0
Range: 123-123

Valid cases: 0
Invalid: 0

Sample swabbing district (S_COLL_D)
File: INSPIRE.PEACH.IDSR.UG.2021.CDM.V1

Overview

Type: Discrete
Format: character
Width: 3

Valid cases: 0
Invalid: 0

Sample collection place (S_COLL)
File: INSPIRE.PEACH.IDSR.UG.2021.CDM.V1

Overview

Type: Discrete
Format: numeric
Width: 10
Decimals: 0
Range: 123-123

Valid cases: 0
Invalid: 0

Reporting facility (H_FACI)
File: INSPIRE.PEACH.IDSR.UG.2021.CDM.V1

Overview

Type: Discrete
Format: character
Width: 3

Valid cases: 0
Invalid: 0

Point of entry (P_ENT)
File: INSPIRE.PEACH.IDSR.UG.2021.CDM.V1

Overview

Type: Discrete
Format: character
Width: 3

Valid cases: 0
Invalid: 0

Who is being tested (W_TST)
File: INSPIRE.PEACH.IDSR.UG.2021.CDM.V1

Overview

Type: Discrete
Format: numeric
Width: 10
Decimals: 0

Valid cases: 0
Invalid: 0

Traveler going in or out of uganda (TRAV)
File: INSPIRE.PEACH.IDSR.UG.2021.CDM.V1

Overview

Type: Discrete
Format: numeric
Width: 10
Decimals: 0
Range: 123-123

Valid cases: 0
Invalid: 0

Reason for health worker test (R_HW_TST)
File: INSPIRE.PEACH.IDSR.UG.2021.CDM.V1

Overview

Type: Discrete
Format: numeric
Width: 10
Decimals: 0
Range: 123-123

Valid cases: 0
Invalid: 0

Health worker facility (HW_FAC)
File: INSPIRE.PEACH.IDSR.UG.2021.CDM.V1

Overview

Type: Discrete
Format: character
Width: 3

Valid cases: 0
Invalid: 0

Isolated/Quarantined testing date (IQ_D_TST)
File: INSPIRE.PEACH.IDSR.UG.2021.CDM.V1

Overview

Type: Discrete
Format: numeric
Width: 10
Decimals: 0
Range: 123-123

Valid cases: 0
Invalid: 0

Date of birth (DOB)
File: INSPIRE.PEACH.IDSR.UG.2021.CDM.V1

Overview

Type: Discrete
Format: character
Width: 11

Valid cases: 0

Estimated age in years (AGE_Y)
File: INSPIRE.PEACH.IDSR.UG.2021.CDM.V1

Overview

Type: Discrete
Format: numeric
Width: 10
Decimals: 0
Range: 123-123

Valid cases: 0
Invalid: 0

Estimated age in months (AGE_M)
File: INSPIRE.PEACH.IDSR.UG.2021.CDM.V1

Overview

Type: Discrete
Format: numeric
Width: 10
Decimals: 0
Range: 123-123

Valid cases: 0
Invalid: 0

sex (SEX)
File: INSPIRE.PEACH.IDSR.UG.2021.CDM.V1

Overview

Type: Discrete
Format: numeric
Width: 10
Decimals: 0
Range: 123-123

Valid cases: 0
Invalid: 0

Nationality (NATL)
File: INSPIRE.PEACH.IDSR.UG.2021.CDM.V1

Overview

Type: Discrete
Format: numeric
Width: 10
Decimals: 0
Range: 123-123

Valid cases: 0
Invalid: 0

Reporting sub county (SUB_COUNT)
File: INSPIRE.PEACH.IDSR.UG.2021.CDM.V1

Overview

Type: Discrete
Format: numeric
Width: 10
Decimals: 0
Range: 123-123

Valid cases: 0
Invalid: 0

Reporting district (REPR_DIST)
File: INSPIRE.PEACH.IDSR.UG.2021.CDM.V1

Overview

Type: Discrete
Format: numeric
Width: 10
Decimals: 0
Range: 123-123

Valid cases: 0
Invalid: 0

Truck destination (TRK_DEST)
File: INSPIRE.PEACH.IDSR.UG.2021.CDM.V1

Overview

Type: Discrete
Format: character
Width: 3

Valid cases: 0
Invalid: 0

Vaccinated vaccinated against covid-19 (VAC)
File: INSPIRE.PEACH.IDSR.UG.2021.CDM.V1

Overview

Type: Discrete
Format: numeric
Width: 10
Decimals: 0
Range: 123-123

Valid cases: 0
Invalid: 0

Name of covid-19 vaccine (VACC_TYP_VAC)
File: INSPIRE.PEACH.IDSR.UG.2021.CDM.V1

Overview

Type: Discrete
Format: numeric
Width: 10
Decimals: 0
Range: 123-123

Valid cases: 0
Invalid: 0

Number of doses (N_DOSE)
File: INSPIRE.PEACH.IDSR.UG.2021.CDM.V1

Overview

Type: Discrete
Format: numeric
Width: 10
Decimals: 0
Range: 123-123

Valid cases: 0
Invalid: 0

Date of last dose (D_LST_DOSE)
File: INSPIRE.PEACH.IDSR.UG.2021.CDM.V1

Overview

Type: Discrete
Format: character
Width: 11

Valid cases: 0

Was patient symptomatic (P_SYMP)
File: INSPIRE.PEACH.IDSR.UG.2021.CDM.V1

Overview

Type: Discrete
Format: numeric
Width: 10
Decimals: 0
Range: 123-123

Valid cases: 0
Invalid: 0

Date of first symptoms (D_ONSE)
File: INSPIRE.PEACH.IDSR.UG.2021.CDM.V1

Overview

Type: Discrete
Format: character
Width: 11

Valid cases: 0

Presenting symptoms (PRES_SYMP)
File: INSPIRE.PEACH.IDSR.UG.2021.CDM.V1

Overview

Type: Discrete
Format: numeric
Width: 10
Decimals: 0
Range: 123-123

Valid cases: 0
Invalid: 0

Underlying conditions (U_CONDS)
File: INSPIRE.PEACH.IDSR.UG.2021.CDM.V1

Overview

Type: Discrete
Format: numeric
Width: 10
Decimals: 0
Range: 123-123

Valid cases: 0
Invalid: 0

Specific underlying conditions (U_CONDS_SPEC)
File: INSPIRE.PEACH.IDSR.UG.2021.CDM.V1

Overview

Type: Discrete
Format: numeric
Width: 10
Decimals: 0
Range: 123-123

Valid cases: 0
Invalid: 0

Specimen type (S_TYP)
File: INSPIRE.PEACH.IDSR.UG.2021.CDM.V1

Overview

Type: Discrete
Format: numeric
Width: 10
Decimals: 0
Range: 123-123

Valid cases: 0
Invalid: 0

Date specimen collected (AG)
File: INSPIRE.PEACH.IDSR.UG.2021.CDM.V1

Overview

Type: Discrete
Format: numeric
Width: 10
Decimals: 0
Range: 23022-23022

Valid cases: 0
Invalid: 0

Time Specimen collected (T_S_COLL)
File: INSPIRE.PEACH.IDSR.UG.2021.CDM.V1

Overview

Type: Discrete
Format: numeric
Width: 10
Decimals: 0

Valid cases: 0
Invalid: 0

Test requested (T_RQST)
File: INSPIRE.PEACH.IDSR.UG.2021.CDM.V1

Overview

Type: Discrete
Format: character
Width: 3

Valid cases: 0
Invalid: 0

Was specimen referred? (S_REFER)
File: INSPIRE.PEACH.IDSR.UG.2021.CDM.V1

Overview

Type: Discrete
Format: numeric
Width: 10
Decimals: 0
Range: 123-123

Valid cases: 0
Invalid: 0

Tester's name (TST_NM)
File: INSPIRE.PEACH.IDSR.UG.2021.CDM.V1

Overview

Type: Discrete
Format: character
Width: 3

Valid cases: 0
Invalid: 0

Type of test (T_TYP)
File: INSPIRE.PEACH.IDSR.UG.2021.CDM.V1

Overview

Type: Discrete
Format: numeric
Width: 10
Decimals: 0
Range: 123-123

Valid cases: 0
Invalid: 0

Final laboratory result (FIN_LAB_RES)
File: INSPIRE.PEACH.IDSR.UG.2021.CDM.V1

Overview

Type: Discrete
Format: numeric
Width: 10
Decimals: 0
Range: 123-123

Valid cases: 0
Invalid: 0

Date result released (D_RES)
File: INSPIRE.PEACH.IDSR.UG.2021.CDM.V1

Overview

Type: Discrete
Format: character
Width: 11

Valid cases: 0

Time result released (T_RES)
File: INSPIRE.PEACH.IDSR.UG.2021.CDM.V1

Overview

Type: Discrete
Format: numeric
Width: 10
Decimals: 0

Valid cases: 0
Invalid: 0

Remarks (RMK)
File: INSPIRE.PEACH.IDSR.UG.2021.CDM.V1

Overview

Type: Discrete
Format: character
Width: 3

Valid cases: 0
Invalid: 0

Additional test required (A_TST_R)
File: INSPIRE.PEACH.IDSR.UG.2021.CDM.V1

Overview

Type: Discrete
Format: numeric
Width: 10
Decimals: 0
Range: 123-123

Valid cases: 0
Invalid: 0

Additional test type (A_TST_T)
File: INSPIRE.PEACH.IDSR.UG.2021.CDM.V1

Overview

Type: Discrete
Format: numeric
Width: 10
Decimals: 0
Range: 123-123

Valid cases: 0
Invalid: 0

Additional test date (A_TST_D)
File: INSPIRE.PEACH.IDSR.UG.2021.CDM.V1

Overview

Type: Discrete
Format: character
Width: 11

Valid cases: 0

Unique identifier (EPID_NO)
File: INSPIRE.PEACH.IDSR.KE.2021.CDM.V1

Overview

Type: Discrete
Format: numeric
Width: 10
Decimals: 0
Range: 123-123

Valid cases: 0
Invalid: 0

Nationality (NATL)
File: INSPIRE.PEACH.IDSR.KE.2021.CDM.V1

Overview

Type: Discrete
Format: numeric
Width: 10
Decimals: 0
Range: 123-123

Valid cases: 0
Invalid: 0

Date form received at national level (D_F_REC)
File: INSPIRE.PEACH.IDSR.KE.2021.CDM.V1

Overview

Type: Discrete
Format: character
Width: 11

Valid cases: 0

Reporting health facility (H_FACI)
File: INSPIRE.PEACH.IDSR.KE.2021.CDM.V1

Overview

Type: Discrete
Format: character
Width: 3

Valid cases: 0
Invalid: 0

Type of Health facility (T_H_FACI)
File: INSPIRE.PEACH.IDSR.KE.2021.CDM.V1

Overview

Type: Discrete
Format: character
Width: 3

Valid cases: 0
Invalid: 0

Reporting sub county (SUB_COUNT)
File: INSPIRE.PEACH.IDSR.KE.2021.CDM.V1

Overview

Type: Discrete
Format: numeric
Width: 10
Decimals: 0
Range: 123-123

Valid cases: 0
Invalid: 0

Reporting county (COUNT)
File: INSPIRE.PEACH.IDSR.KE.2021.CDM.V1

Overview

Type: Discrete
Format: numeric
Width: 10
Decimals: 0
Range: 123-123

Valid cases: 0
Invalid: 0

Type of reporting disease condition (TYP_REPR_DISE_COND)
File: INSPIRE.PEACH.IDSR.KE.2021.CDM.V1

Overview

Type: Discrete
Format: numeric
Width: 10
Decimals: 0
Range: 123-123

Valid cases: 0
Invalid: 0

sex (SEX)
File: INSPIRE.PEACH.IDSR.KE.2021.CDM.V1

Overview

Type: Discrete
Format: numeric
Width: 10
Decimals: 0
Range: 123-123

Valid cases: 0
Invalid: 0

Date of birth (DOB)
File: INSPIRE.PEACH.IDSR.KE.2021.CDM.V1

Overview

Type: Discrete
Format: character
Width: 11

Valid cases: 0

Estimated age in years (AGE_Y)
File: INSPIRE.PEACH.IDSR.KE.2021.CDM.V1

Overview

Type: Discrete
Format: numeric
Width: 10
Decimals: 0
Range: 123-123

Valid cases: 0
Invalid: 0

Estimated age in months (AGE_M)
File: INSPIRE.PEACH.IDSR.KE.2021.CDM.V1

Overview

Type: Discrete
Format: numeric
Width: 10
Decimals: 0
Range: 123-123

Valid cases: 0
Invalid: 0

Estimated age in days (AGE_D)
File: INSPIRE.PEACH.IDSR.KE.2021.CDM.V1

Overview

Type: Discrete
Format: numeric
Width: 10
Decimals: 0
Range: 123-123

Valid cases: 0
Invalid: 0

residence (RES)
File: INSPIRE.PEACH.IDSR.KE.2021.CDM.V1

Overview

Type: Discrete
Format: numeric
Width: 10
Decimals: 0
Range: 123-123

Valid cases: 0
Invalid: 0

Date of onset of illness (D_ONSE)
File: INSPIRE.PEACH.IDSR.KE.2021.CDM.V1

Overview

Type: Discrete
Format: character
Width: 11

Valid cases: 0

Date seen at facility (D_SEEN_FACI)
File: INSPIRE.PEACH.IDSR.KE.2021.CDM.V1

Overview

Type: Discrete
Format: character
Width: 11

Valid cases: 0

Date Health Facility Notified sub county level (D_NOT_SUBC)
File: INSPIRE.PEACH.IDSR.KE.2021.CDM.V1

Overview

Type: Discrete
Format: character
Width: 11

Valid cases: 0

Hospitalized (HOSP)
File: INSPIRE.PEACH.IDSR.KE.2021.CDM.V1

Overview

Type: Discrete
Format: numeric
Width: 10
Decimals: 0
Range: 123-123

Valid cases: 0
Invalid: 0

Date of admission (D_ADM)
File: INSPIRE.PEACH.IDSR.KE.2021.CDM.V1

Overview

Type: Discrete
Format: character
Width: 11

Valid cases: 0

Inpatient/outpatient number (IP_OP_NO)
File: INSPIRE.PEACH.IDSR.KE.2021.CDM.V1

Overview

Type: Discrete
Format: numeric
Width: 10
Decimals: 0
Range: 123-123

Valid cases: 0
Invalid: 0

Diagnosis (DIAG)
File: INSPIRE.PEACH.IDSR.KE.2021.CDM.V1

Overview

Type: Discrete
Format: character
Width: 3

Valid cases: 0
Invalid: 0

Means of Diagnosis (M_DIAG)
File: INSPIRE.PEACH.IDSR.KE.2021.CDM.V1

Overview

Type: Discrete
Format: numeric
Width: 10
Decimals: 0
Range: 123-123

Valid cases: 0
Invalid: 0

Vaccination History (VACC_HIST)
File: INSPIRE.PEACH.IDSR.KE.2021.CDM.V1

Overview

Type: Discrete
Format: numeric
Width: 10
Decimals: 0
Range: 123-123

Valid cases: 0
Invalid: 0

Number of doses (N_DOSE)
File: INSPIRE.PEACH.IDSR.KE.2021.CDM.V1

Overview

Type: Discrete
Format: numeric
Width: 10
Decimals: 0

Valid cases: 0
Invalid: 0

Vaccination given last two months (VACC_L_T_MON)
File: INSPIRE.PEACH.IDSR.KE.2021.CDM.V1

Overview

Type: Discrete
Format: numeric
Width: 10
Decimals: 0
Range: 123-123

Valid cases: 0
Invalid: 0

Date vaccination given last two months (D_VACC_L_T_MON)
File: INSPIRE.PEACH.IDSR.KE.2021.CDM.V1

Overview

Type: Discrete
Format: character
Width: 11

Valid cases: 0

Status of the Patient (CASE_F_CLASS)
File: INSPIRE.PEACH.IDSR.KE.2021.CDM.V1

Overview

Type: Discrete
Format: numeric
Width: 10
Decimals: 0
Range: 123-123

Valid cases: 0
Invalid: 0

Date of onset of paralysis (D_ONSE_PARA)
File: INSPIRE.PEACH.IDSR.KE.2021.CDM.V1

Overview

Type: Discrete
Format: character
Width: 11

Valid cases: 0

Paralysis signs and symptoms (PARA_SYMP)
File: INSPIRE.PEACH.IDSR.KE.2021.CDM.V1

Overview

Type: Discrete
Format: numeric
Width: 10
Decimals: 0
Range: 123-123

Valid cases: 0
Invalid: 0

Paralysis resenting symptoms (PARA_SYMP_PRES)
File: INSPIRE.PEACH.IDSR.KE.2021.CDM.V1

Overview

Type: Discrete
Format: numeric
Width: 10
Decimals: 0
Range: 123-123

Valid cases: 0
Invalid: 0

Site of paralysis (SITE_PARA)
File: INSPIRE.PEACH.IDSR.KE.2021.CDM.V1

Overview

Type: Discrete
Format: numeric
Width: 10
Decimals: 0

Valid cases: 0
Invalid: 0

Where was the baby delivered (DELV)
File: INSPIRE.PEACH.IDSR.KE.2021.CDM.V1

Overview

Type: Discrete
Format: numeric
Width: 10
Decimals: 0
Range: 123-123

Valid cases: 0
Invalid: 0

Health facility baby was delivered (H_FACI_DELV)
File: INSPIRE.PEACH.IDSR.KE.2021.CDM.V1

Overview

Type: Discrete
Format: character
Width: 3

Valid cases: 0
Invalid: 0

Was cord cut with sterile blade (CORD_ST)
File: INSPIRE.PEACH.IDSR.KE.2021.CDM.V1

Overview

Type: Discrete
Format: numeric
Width: 10
Decimals: 0
Range: 123-123

Valid cases: 0
Invalid: 0

How was cord stump treated (CORD_TREAT)
File: INSPIRE.PEACH.IDSR.KE.2021.CDM.V1

Overview

Type: Discrete
Format: character
Width: 3

Valid cases: 0
Invalid: 0

how old in days symptoms began (AGE_D_SYMP)
File: INSPIRE.PEACH.IDSR.KE.2021.CDM.V1

Overview

Type: Discrete
Format: numeric
Width: 10
Decimals: 0
Range: 123-123

Valid cases: 0
Invalid: 0

Did baby suck normally at birth (SUCK_N)
File: INSPIRE.PEACH.IDSR.KE.2021.CDM.V1

Overview

Type: Discrete
Format: numeric
Width: 10
Decimals: 0
Range: 123-123

Valid cases: 0
Invalid: 0

Case confirmed neonatal tetanus (N_TT)
File: INSPIRE.PEACH.IDSR.KE.2021.CDM.V1

Overview

Type: Discrete
Format: numeric
Width: 10
Decimals: 0
Range: 123-123

Valid cases: 0
Invalid: 0

Treated at health facility (TX_HC)
File: INSPIRE.PEACH.IDSR.KE.2021.CDM.V1

Overview

Type: Discrete
Format: numeric
Width: 10
Decimals: 0
Range: 123-123

Valid cases: 0
Invalid: 0

Is the mother alive (MOM_ALIVE)
File: INSPIRE.PEACH.IDSR.KE.2021.CDM.V1

Overview

Type: Discrete
Format: numeric
Width: 10
Decimals: 0
Range: 123-123

Valid cases: 0
Invalid: 0

Did case response for the mother take place (CR_MOM)
File: INSPIRE.PEACH.IDSR.KE.2021.CDM.V1

Overview

Type: Discrete
Format: numeric
Width: 10
Decimals: 0
Range: 123-123

Valid cases: 0
Invalid: 0

Case response in community (CR_COMM)
File: INSPIRE.PEACH.IDSR.KE.2021.CDM.V1

Overview

Type: Discrete
Format: numeric
Width: 10
Decimals: 0
Range: 123-123

Valid cases: 0
Invalid: 0

Presence of fever (FEVER)
File: INSPIRE.PEACH.IDSR.KE.2021.CDM.V1

Overview

Type: Discrete
Format: numeric
Width: 10
Decimals: 0
Range: 123-123

Valid cases: 0
Invalid: 0

Date of onset of rash (D_ONSE_RASH)
File: INSPIRE.PEACH.IDSR.KE.2021.CDM.V1

Overview

Type: Discrete
Format: character
Width: 11

Valid cases: 0

Type of rash (TYP_RASH)
File: INSPIRE.PEACH.IDSR.KE.2021.CDM.V1

Overview

Type: Discrete
Format: numeric
Width: 10
Decimals: 0
Range: 123-123

Valid cases: 0
Invalid: 0

Patient visited for contact investigation (CONT_INVE)
File: INSPIRE.PEACH.IDSR.KE.2021.CDM.V1

Overview

Type: Discrete
Format: numeric
Width: 10
Decimals: 0
Range: 123-123

Valid cases: 0
Invalid: 0

Date of contact investigation (D_CONT_INVE)
File: INSPIRE.PEACH.IDSR.KE.2021.CDM.V1

Overview

Type: Discrete
Format: character
Width: 11

Valid cases: 0

Case epidemiologically linked to lab confirmed case (LAB_CON_CASE)
File: INSPIRE.PEACH.IDSR.KE.2021.CDM.V1

Overview

Type: Discrete
Format: numeric
Width: 10
Decimals: 0
Range: 123-123

Valid cases: 0
Invalid: 0

Was specimen collected (S_COLL)
File: INSPIRE.PEACH.IDSR.KE.2021.CDM.V1

Overview

Type: Discrete
Format: numeric
Width: 10
Decimals: 0
Range: 123-123

Valid cases: 0
Invalid: 0

Date Specimen collected (D_S_COLL)
File: INSPIRE.PEACH.IDSR.KE.2021.CDM.V1

Overview

Type: Discrete
Format: character
Width: 11

Valid cases: 0

Specimen Type (S_TYP)
File: INSPIRE.PEACH.IDSR.KE.2021.CDM.V1

Overview

Type: Discrete
Format: numeric
Width: 10
Decimals: 0
Range: 123-123

Valid cases: 0
Invalid: 0

Date Specimen sent to lab (D_S_SENT_LAB)
File: INSPIRE.PEACH.IDSR.KE.2021.CDM.V1

Overview

Type: Discrete
Format: character
Width: 11

Valid cases: 0

Name of lab (NAME_LAB)
File: INSPIRE.PEACH.IDSR.KE.2021.CDM.V1

Overview

Type: Discrete
Format: character
Width: 3

Valid cases: 0
Invalid: 0

Received lab results (LAB_RESU_R)
File: INSPIRE.PEACH.IDSR.KE.2021.CDM.V1

Overview

Type: Discrete
Format: numeric
Width: 10
Decimals: 0
Range: 123-123

Valid cases: 0
Invalid: 0

Received lab results not received (LAB_RESU_NR)
File: INSPIRE.PEACH.IDSR.KE.2021.CDM.V1

Overview

Type: Discrete
Format: numeric
Width: 10
Decimals: 0
Range: 123-123

Valid cases: 0
Invalid: 0

Final laboratory result (FIN_LAB_RES)
File: INSPIRE.PEACH.IDSR.KE.2021.CDM.V1

Overview

Type: Discrete
Format: numeric
Width: 10
Decimals: 0
Range: 123-123

Valid cases: 0
Invalid: 0
